# Skin Diseases and Syphilis

**Published:** 1895-10

**Authors:** 


### SKIN DISEASES AND SYPHILIS.

EDITED BY DR. M. B. HUTCHINS.

### TREATMENT OF PSORIASIS BY THE EXTRACT OF THE THYROID BODY.

The few attempts which have been made in France by this method of treatment have given the most variable results. For my own part, I have seen it result favorably in only one instance, in a patient in private practice who had a psoriasis proving most rebellious to all local treatment, and in which the eruption was greatly improved under the influence of the ingestion of pastilles of thyroid extract. When he ceased to make use of the remedy the eruption took on all of its original intensity, but retroceded when he began its use again. On the other hand, I had entire unsuccess, and in fact accidents of quite a serious nature, so that I was obliged to interdict the use of this substance for my patients. Dr. Messeau has just caused to appear in the "Transactions of the Policlinique Dermatologique de Bordeaux," directed by Dr. Dubreuilh, a most interesting memoir, in which he reviews the principal works published upon the question, and in which he reports four observations collected by himself from the service of Dr. Dubreuilh. In the first two cases the treatment was carried out by means of pastilles of the thyroid body. It was continued for about a month, without the least result in cases of severe psoriasis. In the third case (very light and recent psoriasis) the extract of thyroid juice was used, and at the end of a month and a half the amelioration was only very mediocre, when local treatment was instituted. In the fourth case (psoriasis of severe and rebellious type), the natural thyroid body was given fresh. The first ingestion determined a violent malaise, with febrile movement and violent diarrhea; at three other times the attempt was made to give thyroid body, but the same general symptoms were each time reproduced and it could not be continued. The author concludes that we cannot regard the treatment by thyroid body as a specific for psoriasis.—DR. L. BROCQ, Paris, in *Journal Gen.-Ur. and Cutaneous Diseases*, August, 1895.

The disease known as myxedema was the first in which the thyroid gland or its extract was administered. The disease was found to arise from a deficiency in function or structure of the thyroid gland, and dosing with the glands of some of the food animals seemed to supply the missing element of nutrition, clearing up all the distressing symptoms and rendering the skin and its appendages again practically normal.

Dermatologists were led to use the treatment in various other skin diseases, and the scaly psoriasis seemed especially promising, but as a whole they have found little encouragement in its effects. Thyroid glands of calves and sheep were first used, later extracts.

H.

---

### CLINICAL NOTES.

(1) Case of a man, aged sixty-nine. Epithelioma developing as result of razor cut in an ordinary, skin-colored nævus in front of the left ear. Disease of eight months' duration. Microscope confirmed the diagnosis. Cured by excision. H.

(2) Mulatto—male. Papulo-pustular syphilitic eruption. Intense iritis in right eye.

"Mixed" treatment internally, atropia sulphate solution in the eye, and periorbital inunctions of mercurial ointment. Rapid recovery. H.

(3) Mulatto—girl, aged sixteen. No evidence of syphilitic infection save in lower margin of the umbilicus, where a denuded, bean-sized papule was present for two months. A few weeks after the appearance of the above lesion "mucous patches" developed in the mouth and throat, and the vulvar and perineal regions became covered with large *condylomata lata*. The patient's vitality is very much below par. This appears to be a case of "initial (syphilitic) lesion" of the umbilicus. H.

(4) Case of a one-legged, middle-aged negro man. Only evidence of initial lesion was a fissured infiltration in the center of the lower lip, on the carmine border. Later numerous mucous patches in the mouth, followed in a few weeks by a scant papular (lentiginous) eruption on the neck. General adenopathy marked from the first observation of this case. H.

(5) A case of ichthyosis hystrix (congenital) in a negro boy of fourteen. The greater part of the surface, exclusive of palms, soles, last phalanges, face, neck, and genitals, nearly symmetrically involved. The affected skin being either hard and dry, scaly, or, as the greater part, the skin was much thickened in its upper layers and studded with large, pin-head sized papules, rather flat, capped with a horny mass. These papules, or verrucæ followed quite accurately the natural lines of the skin, which were well defined. The boy was dull, stupid, and rather illy developed. H.

NOTE.—The cases mentioned in these notes will come chiefly from my clinic at the Atlanta Medical College, though others of interest may be taken from my private notes. M. B. H.

---

### TREATMENT OF FURUNCULOSIS.

It is well known that there are rebellious cases of boils in which the analysis of the urine shows neither diabetes of any kind nor albuminuria. None of the internal remedies vaunted hitherto appear to act, and boils or carbuncles succeed each other for months with a tenacity which is truly discouraging. In chronic gouty subjects in whom this furunculosis seems to have replaced the clearly defined accidents of the gout itself, I have tried the use of colchicum. In a gouty subject of forty years, affected for months with furunculosis, the action of the extract of colchicum, given in doses of two or three centigrammes *per diem*, was in a way remarkable. From the sixth day the evolution of the furuncles already existing was arrested, and no new ones appeared. A little surprised at the result, the patient ceased using the colchicum, and at the end of a fortnight several boils showed themselves again. He then renewed the colchicum, and the affection was quickly subdued. These experiences were repeated several times, until the day when the subject, perfectly elated over the power of the colchicum, took it upon himself to continue the remedy for a long time, and then to cease its use gradually. He is at the present time completely cured of his furunculosis. As adjuvant and as local treatment, I prescribed besides daily lotions with camphorated alcohol over the whole body, and over the boils themselves employed Vidal's red plaster (minium, cinnabar, and diachylon). Since this first cure I have given colchicum to several other patients affected with furunculosis, but with varying success. I completely failed in some cases, especially when the furunculosis complicated eczematous eruptions. The colchicum seems, too, to have very little action upon the folliculites of sycosis vulgaris. I reserve the communication of the final results of my observations, with all the necessary details, for a time when I shall have collected sufficient data. For the moment I confine myself to the statement that in certain cases of rebellious furunculosis developing in patients without diabetes and without albuminuria, colchicum administered in sufficient doses has given good results.—DR. BROCQ, Paris, *Journ. Cut. and Gen.-Ur. Dis.*, August, 1895.

---

### SYPHILITIC MUCOUS PATCHES OF THE CONJUNCTIVA.

Dr. Albert Staelin (*Monatshefte für pract. Dermatologie*, vol. xx, No. 1).

This paper forms part of a work on eye affections in syphilitics, which the author is about to publish in conjunction with Dr. Wilbrand, a well-known oculist of Hamburg.

Staelin gives a detailed report of twenty-one cases of this secondary specific affection of the eye found in examining two hundred patients. These figures show that the common view that mucous patches of the conjunctiva are very rare is an erroneous one. In most of his cases specific papules were observed in other parts of the body. The papules of the conjunctiva do not differ in their aspects from those on other mucous membranes, only being smaller, their size varying from that of a pin-head to that of a pea. They have a semicircular shape, and if left alone, they show a tendency to ulcerate. Under specific treatment the nodule disappears entirely, only leaving a red basis, which in most cases likewise disappears. The most common site of the papule is the conjunctiva of the lower lid, and especially the *fornix conjunctivæ*. There being no conjunctival irritation and hardly any subjective symptoms, this affection, which the author found in over ten per cent. of his cases, is very often overlooked.—*Journ. Cut. and Gen.-Ur. Dis.*, August, 1895.

---

### ECZEMA AND ITS NATURE.

Professor Breda, of Padua (*Archiv für Dermat. und Syph.*, 1894, vol. xxix, No. 2).

A well-written and interesting essay in which the author defines eczema as "a superficial, non-contagious, polymorphous, pruriginous, usually chronic dermatitis, which has a tendency to spread and to relapse, and which does not leave any scars."

He specifies, as not belonging to the class of eczema, erythema, eczema marginatum, staphylococcia (Wickham), Paget's disease, dysidrosis, mycosis fungoides (initial stage), tuberculosis cutanea, the different forms of neurodermatitis, keratodermia, eczematization of Brocq and Jacquet, eczema seborrhoicum (Unna), and dermatitis artificialis (caused by external irritations).

Etiologically the author believes more or less in the "herpetismus and arthritismus" as factors, besides which a certain vulnerability of the skin and disturbances of the innervation play an important part.—*Journ. Cut. and Gen.-Ur. Dis.*, August, 1895.

